# Draft Genome Sequence of Antimicrobial-Producing *Clostridium* sp*.* JC272, Isolated from Marine Sediment

**DOI:** 10.1128/genomeA.00650-15

**Published:** 2015-06-11

**Authors:** L. Tushar, T. S. Sasi Jyothsna, C. Sasikala, C. V. Ramana

**Affiliations:** a Department of Plant Sciences, School of Life Sciences, University of Hyderabad, Hyderabad, India; b Centre for Environment, Bacterial Discovery Laboratory, Institute of Science and Technology, J. N. T. University Hyderabad, Hyderabad, India

## Abstract

We announce the draft genome sequence of *Clostridium* sp. JC272, isolated from a sediment sample collected from marine habitats of Gujarat, India. *Clostridium* sp. JC272 is an obligate anaerobe and has the ability to produce antimicrobial compounds. The genome sequence indicates the strain’s capability of producing small peptides (microcins), which are potential novel antibiotics.

## GENOME ANNOUNCEMENT

There is limited information about antibiotics and secondary metabolites produced by obligate anaerobic bacteria. Whole-genome sequences of *Clostridium* spp. have revealed the wide occurrence of biosynthetic gene clusters coding for polyketide synthases (PKS) and nonribosomal peptide synthases (NRPs) involved in antibiotic biosynthesis (1, 2). The discovery of closthioamide, a polythioamide antibiotic from *Clostridium cellulolyticum* (3) widens the search for novel antibiotics from other members of the genus.

*Clostridium* sp. JC272 was isolated from a sediment sample collected from Gujrat, India (23°8′82″N and 69°74′E). Genome sequencing was carried out using the Illumina HiSeq 2000 (Illumina, Inc.) platform. Assembly of the raw sequencing data was performed using MaSuRCA (de Bruijn graph and overlap-layout-consensus). Annotation of the assembled data was performed using the Rapid Annotations using Subsystem Technology (RAST) server (4). For antibiotics and secondary metabolites analysis, the antiSMASH (http://antismash.secondarymetabolites.org) server was used. The proposal of strain JC272 as a new species to the genus *Clostridium* is also evidenced from the species identification tool *SpecI*, which is based on core genome analysis and the results supported strain JC272 as a novel species (5).Whole-genome sequencing of strain JC272 yielded a genome of 3,568,807 bp in length. The calculated G+C mol% of the bacterium was 28.3%. Annotation of the genome consisted of 2,174 coding sequences that included 105 RNA genes. Only one copy of the 16S rRNA gene (1,530 bp) was identified in strain JC272. Annotation reveals the presence of 60 genes related to motility and chemotaxis and 149 genes related to cell wall and capsule formation. Eighty-two genes are involved in the biosynthesis of fatty acid, lipids, and isoprenoids. Based on a KEGG analysis using the RAST server, the genome of strain JC272 showed the presence of 11 genes related to nitrogen metabolism, 54 genes related to phosphorus metabolism, and 6 genes related to potassium metabolism. Moreover, 82 genes related to membrane transport and 66 genes related to respiration are also present in the genome of strain JC272. Isoprenoid biosynthesis occurred through the 2-C-methyl-d-erythritol 4-phosphate (MEP) pathway in strain JC272. Hopanoid biosynthetic pathway genes were not found in the draft genome sequence of strain JC272 or in the available 190 genomes of *Clostridium*.

Strain JC272 has gene clusters responsible for the production of microcins, which are responsible for antimicrobial activity (6, 7). Four small peptides (45, 64, 77, and 66 amino acids) were predicted to be microcins from the draft genome of strain JC272, whereas the nearest genome of *C. bifermentans* ATCC 638 has only two sets of genes producing two small peptides (37 and 77 amino acids). These microcins have a two-component system that comprises a sensory histidine kinase (HK) and its cognate response regulator (RR). The HK gets autophosphorylated followed by phosphorylation of the receiver domain of RR (8). Methanolic extracts of strain JC272 show good antimicrobial activity against *E. coli*, *S. aureus*, and *Bacillus* spp., and work is in progress to validate the microcins.

### Nucleotide sequence accession numbers. 

This whole-genome shotgun project has been deposited at DDBJ/EMBL/GenBank under the accession number LBBT00000000. The version described in this paper is the first version, LBBT01000000.
